# Transcription Regulation of Plastid Genes Involved in Sulfate Transport in Viridiplantae

**DOI:** 10.1155/2013/413450

**Published:** 2013-08-29

**Authors:** Vassily A. Lyubetsky, Alexander V. Seliverstov, Oleg A. Zverkov

**Affiliations:** Institute for Information Transmission Problems (Kharkevich Institute), The Russian Academy of Sciences, Moscow 127994, Russia

## Abstract

This study considers transcription regulation of plastid genes involved in sulfate transport in the parasites of invertebrate (*Helicosporidium* sp.) and other species of the Viridiplantae. A one-box conserved motif with the consensus TAAWATGATT is found near promoters upstream the *cysT* and *cysA* genes in many species. In certain cases, the motif is repeated two or three times.

## 1. Introduction

This study focuses on selected species of the Viridiplantae, particularly, the genus *Helicosporidium* sp. (class Trebouxiophyceae), which comprises green algae parasitizing flies of the species *Simulium jonesi * [1–3]. Plastids of these parasites are a good target for antibiotic treatment, as earlier was shown for apicomplexan parasites of vertebrates (*Toxoplasma gondii* and *Plasmodium *spp. [4]).

The plastome of *Helicosporidium* sp. is relatively small, about 37 kb. Most of the plastome genes encode tRNA, rRNA, ribosomal proteins, and subunits of the bacterial-type RNA polymerase. One of two nonhousekeeping proteins is the CysT subunit of a sulfate ABC transporter.

Sulfate ABC transporters in cyanobacteria and proteobacteria consist of two identical ATP-binding CysA proteins, two transmembrane proteins (CysT and CysW), and a sulfate-binding protein SbpA. In the cyanobacteria *Synechocystis* sp. PCC 6803 [5], genes encoding the sulfate transporter subunits are arranged in a single operon *sbpA-ssr2439-cysT-cysW-cysA*. In cyanobacteria, no data on expression is available for this operon; however, in *Escherichia coli* and some other proteobacteria, genes of the sulfate transporter subunits are known to be regulated in the single operon *cysPTWAM *(further details are given in Discussion).

Plastomes of vascular plants lack genes of the sulfate transport system except for rare instances of *cysT *and *cysA. *However, the green alga *Helicosporidium* sp. retains *cysT*. Plastomes of the rhodophyte *Cyanidium caldarium* and *Cyanidioschyzon merolae* and the cyanelle genome of *Cyanophora paradoxa* lack *cysT* homologues but possess distant homologues of *cysA* presumably involved in the transport of zinc or manganese (further details are given in Results). Similar proteins are involved in the transport of molybdenum, zinc, and manganese and belong to a large family of transporters of ions, sugars, peptides, and more complex organic molecules. For example, the transcription regulation of the *ziaA* gene (encoding a polypeptide similar to a P-type ATPases involved in transporting heavy metals) is described in the cyanobacterium *Synechocystis PCC 6803* [6].

The sulfate transport in plastids is necessary for the synthesis of many sulfur-containing compounds. For example, in *Spinacia oleracea*, the lack of sulfates leads to considerable changes in the expression of cysteine synthesis genes [7]. Also, plastids of many algae synthesize thiamine and other sulfur-containing compounds. For example, the lipoic acid is synthesized in plastids of apicomplexan parasites [8].

In this paper, we consider the expression regulation of *cysT* and *cysA* in Viridiplantae, in particular, *Helicosporidium *sp. and *Pycnococcus provasolii*, where *cysT* is present and *cysA* is absent.

In proteobacteria, the regulation mechanism of transcription initiation of *cysA* and* cysT *is known. The CysB protein is a transcription factor of the LysR family and acts as a tetramer. This protein binds DNA upstream the −35 box of a promoter and activates transcription initiation of many operons. For proteobacteria *Salmonella typhimurium*, *Escherichia coli* [9], and *Klebsiella aerogenes* [10], the CysB activation of *cysPTWAM*, *cysK*, *cysJIH*, *cysDNC*, *sbp*, and L-cysteine transport genes, as well as CysB autorepression is described in detail. Binding of CysB to DNA is not directly dependent on sulfate concentration but requires high concentrations of acetylserine. Also, proteobacteria lack a distinct binding motif for CysB.

## 2. Materials and Methods

The list of species is given in Table 1. Genomes were obtained from GenBank, NCBI. Clustering of proteins was performed using the method described in [11, 12].

An original algorithm from [13, 14] was employed to search for bacterial-type promoters. Relevant promoter sequences and the evolutionary impact of DNA point mutations on polymerase binding affinity are described in [15, 16]; experimental evidence was obtained using the* psbA* promoter of *Sinapis alba* [17].

A novel method based on clique search in a multipartite graph [18] was used to identify conserved motifs. In current modification of the method, the nucleotide similarity was estimated accounting for the GC content in plastid DNA. Namely, if the average GC-rate was  *p*, the additive contribution for a mismatch at any position in the calculation of the distance between two words of equal lengths was (1 − *p*) for a A-T pair, *p* for a C-G pair, and 1/2 for a S-W pair, where S = {C, G} and W = {A, T}. A large-scale search for binding sites was performed using formulas from [19]. Protein alignments were constructed with ClustalW v. 2.0.3 [20].

## 3. Results

### 3.1. Analysis of the Domain Structure and Phylogenetic Classification of Proteins

The *cysT* gene is present in the following species of Viridiplantae: Chlorophyta (*Bryopsis hypnoides, Nephroselmi solivacea, Pycnococcus provasolii, Chlorella variabilis, Chlorella vulgaris, Coccomyxa subellipsoidea C-169, Helicosporidium *sp. ex* Simulium jonesii, Leptosira terrestris,* and *Parachlorella kessleri*), Streptophyta (*Chlorokybus atmophyticus, Mesostigma viride, *and *Zygnema circumcarinatum*), Bryophyta (*Anthoceros formosae, Marchantia polymorpha, *and *Aneura mirabilis*), and *Ptilidium pulcherrimum* (*A.m. *and* P.p.* are pseudogenes) [21].

**Figure 1:**
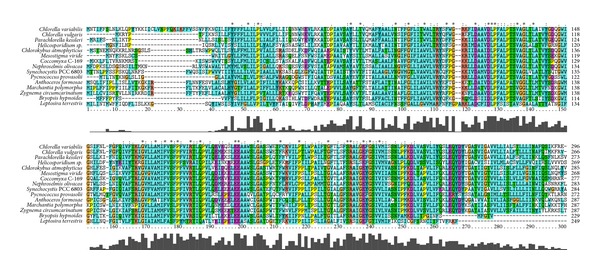
A multiple alignment of CysT orthologs from the cyanobacterium *Synechocystis* sp. PCC 6803 and plastids of the Viridiplantae. Conservativity is shown above and below the columns.

CysT proteins are conserved across green algae, cyanobacteria, and proteobacteria and function as the transmembrane domain of the ABC transporter (Figure 1). Short N-terminus regions of these proteins can vary. Another exception is CysT in *Bryopsis hypnoides* and *Leptosira terrestris* that have truncated C-termini.

Cyanobacteria possess strong potential orthologs of *cysT* in the Viridiplantae. Cyanobacterial CysT is considered to be ancestral.

As CysA and CysT functions are linked, *cysA *and *cysT *normally either coexist or are absent in plastids of all Viridiplantae with the exception of* Helicosporidium* sp. and *Pycnococcus provasolii*. (Note that the *cysA* ortholog in *Marchantia polymorpha* is named *mbpX*.) Viridiplantae species lacking *cysA* and *cysT* are closely related to the species that contain both genes [11, 22]. Unexpectedly, *cysA* and *cysT* are present in Bryophyta while they are absent in many highly organized algae close to land plants (*Chaetosphaeridium globosum*, *Chara vulgaris*, and *Staurastrum punctulatum*). They are also absent in plastomes of *Physcomitrella patens *and all vascular plants. In green algae, these genes are mainly present in the class Trebouxiophyceae (genera *Chlorella*, *Coccomyxa*,* Helicosporidium*, *Leptosira*, and *Parachlorella*).

**Figure 2:**
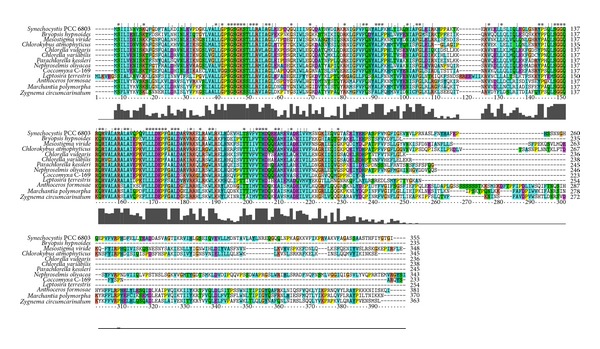
A multiple alignment of CysA orthologs from the cyanobacterium *Synechocystis* sp. PCC 6803 and plastids of the Viridiplantae.

Sequences of plastid CysA and their orthologs from cyanobacterium are well aligned (Figure 2). CysA in all Viridiplantae has a highly conservative N-terminus domain which is typical for the ATP-binding cassette of ABC transporters. In all studied Chlorophyta, except for *Nephroselmis olivacea, *this protein possesses a short C-terminus. Conversely, in the Streptophyta, *Nephroselmis olivacea*, and cyanobacteria, C-termini are long and conservative. In *Mesostigma viride* and* Chlorokybus atmophyticus*, this domain is homologous to the TOBE domain involved in sulfate binding [23]. According to the Pfam 26.0 database [24], *e*-value for this domain is 0.0017 in* M. viride* and 0.00007 in *Ch. atmophyticus. *Other plastid-encoded proteins, although also being well conserved, have a lower score for this domain. There is no sulfate-binding CysP (SbpA) subunit in plastids which could indicate that *cysP* is located in the nucleus.

Outside of the Viridiplantae group, CysA orthologs are encoded in plastids of *Cyanidium caldarium* (NP_045139.1), *Cyanidioschyzon merolae* (NP_848950.1), and* Cyanophora paradoxa* (NP_043273.1). Interestingly, cyanobacteria CysA orthologs differ from plastid CysA orthologs. The NP_043273.1 protein of *C. paradoxa* is an ortholog for the substrate-binding subunit of a zinc or manganese transporter.

### 3.2. Analysis of the Genomic Context

Genes upstream and downstream *cysT* and *cysA* are listed in Table 2. The *rpl32 *gene located upstream *cysT *encodes the ribosomal protein L32 and in most cases belongs to the same DNA strand as *cyst*; refer to Table 2.  *Pycnococcus provasolii*, *Bryopsis hypnoides*, *Helicosporidium* sp., *Leptosira terrestris*, and *Zygnema circumcarinatum* have different gene configurations of this loci; refer to Table 2. Only in *Chlorokybus atmophyticus*,* rpl32* is both upstream of *cysT* and belongs to a different strand than* cysT*. In most cases, the intergenic region upstream the *cysT* gene is quite long. The *ycf1 *gene is present downstream *cysT* in most algae. A few other genes are found downstream *cysT*: tRNA in Bryophyta and the alga *Leptosira terrestris*;* rpl21 *(L21 protein) in the alga* Zygnema circumcarinatum*; *rpoA* (alpha subunit of bacterial-type RNA polymerase) in* Bryopsis hypnoides*; refer to Table 2.

The *accD* gene is located upstream *cysA* and belongs to the same DNA strand in Trebouxiophyceae algae, except for *Leptosira terrestris*. In *Nephroselmis olivacea*, however, a tRNA gene upstream *cysA* is on the complementary strand. In *Bryopsis hypnoides, ccsA* is upstream *cysA, *and the intergenic region is very short. In Streptophyta, *cysA* is surrounded by tRNA genes, and they often belong to the complementary strand which suggests the presence of a promoter directly in the upstream region of *cysA*.

### 3.3. Searching for Bacterial Type Promoters

Only two candidate bacterial-type promoters are found in 5′-leader regions of the considered genes; refer to Table 2. The exception is the *cysA* gene in *Anthoceros formosae*, for which we detect three potential promoters of similar quality. In *Chlorella vulgaris,* the single promoter candidate is located upstream *cysA *and has the unusual −35 box, AAGAAA, which was the reason for its rejection. However, in *Ch*. *variabilis,* a good potential promoter is detected in the upstream region of this gene with a TG-extension of the −10 box. Promoters were not found in the upstream regions of either *cysA *or *cysT* in *Nephroselmis olivacea*, *Pycnococcus provasolii*,* Bryopsis hypnoides*, *Leptosira terrestris*, *Aneura mirabilis,* and *Ptilidium pulcherrimum.* Promoters were not found in the upstream region of *cysA* in* Chlorokybus atmophyticus* and in the upstream region of *cysT* in *Zygnema circumcarinatum. *We speculate that in these cases these genes are transcribed as a part of an operon or by an RNA polymerase of the phage type.

### 3.4. Searching for the Conservative Motif

**Figure 3:**
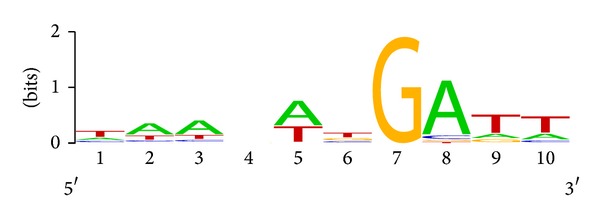
A LOGO nucleotide frequency profile for 10 positions of the found motif.

Transcription regulation of plastid genes involved in the sulfate transport in the parasites of invertebrate (*Helicosporidium* sp.) and in other species of the Viridiplantae is considered. A one-box conserved motif with the consensus TAAWATGATT is found near the promoters in the upstream regions of the *cysT* and *cysA* genes in many species. In some cases, the motif is repeated two or three times. In the upstream region of the *cysA *promoter in alga *C. subellipsoidea* C-169, however, the entire motif is repeated twice and is supplemented with its partial repeat at the 5′-terminus. The motif is not present near the promoters in *Chlorokybus atmophyticus* and *Marchantia polymorpha*. Deviations from the motif consensus are often the same in the same taxonomic lineage, which may reflect the variability of the transcription factor between lineages. The consensus was obtained from multiple alignments of 28 regions upstream two genes in 9 species (*Coccomyxa subellipsoidea*, *Chlorella variabilis*, *Chlorella vulgaris*, *Helicosporidium*,* Parachlorella kessleri*, *Mesostigma viride*, *Chlorokybus atmophyticus*, *Zygnema circumcarinatum*, and *Anthoceros formosae*). The LOGO profile of this motif is shown in Figure 3.

In most species, the motif is found upstream the −35 box or is overlapping the promoter. In the *cysA *upstream region in *Zygnema circumcarinatum* and *Anthoceros formosae*, the motif is detected between −35 and −10 boxes or is overlapping the −10 box of the promoter.

## 4. Discussion

We believe that the found motif represents binding sites of a transcription factor because of its positional linking with the promoter. The variable distance between the motif and the promoters suggests a repressor role of a putative transcription factor. Repeating of the motif is typically associated with a cooperative factor binding. This cooperativity can compensate for the motif variability, which is the case of *Coccomyxa subellipsoidea*.

The motif sequence confirms that *Helicosporidium* sp. belongs to the class Trebouxiophyceae. Its conservativity emphasizes the importance of *cysT *in plastids of parasites and suggests its key importance in understanding the role of the plastids in virulence. Indeed, plastids often synthesize many chemicals, which are usually provided by mitochondria [7, 25].

In *Leptosira terrestris, cysA* and *cysT* are not predicted to have the regulatory sites, unlike other Trebouxiophyceae and their close relatives, which suggests a shift in the transporter (consisting of CysA, CysT, and CysP subunits) specificity in *Leptosira*. This observation conforms with considerable changes in the CysA sequence in *Leptosira*. The lack of regulatory sites in *Nephroselmis olivacea*, *Pycnococcus provasolii*,* Bryopsis hypnoides*, and *Marchantia polymorpha *may suggest a demising role of the protein, which is consistent with the loss or pseudogene nature of *cysA* and *cysT *in *Aneura mirabilis* and *Ptilidium pulcherrimum*. The absence of bacterial-type promoters upstream *cysA* and *cysT* is often associated with changes in genes order on the chromosome. This effect may be explained by the *de novo* formation of phage-type promoters (possibly activated by another factor), or the inclusion of *cysA* or *cysT* in another operons. In general, the sulfate transport can be regulated by changing the expression level of a nuclear encoded sulfate-binding domain CysP (SbpA).

CysB binding sites in proteobacteria [9, 10] differ considerably from binding sites of a putative factor that we predicted for *cysT* and *cysA*. However, the two most conserved motif positions in plastids coincide with the two conserved positions in experimentally characterized sites upstream *cysPTWAM*, *cysK*, *cysJIH*, *cysDNC*, *sbp*, and *cysB* in proteobacteria. This evidence is however insufficient to establish the identity of our predicted motif and the CysB binding sites in proteobacteria. In *E. coli,* both proteins CysT and CysW consist of transmembrane domains that are very close to each other. Their genes belong to the sulfate transport operon *cysPTWAM*. But the CysW protein is absent in plastids. We hypothesize that CysW is replaced by the second CysT copy in plastids. The *cysT* and* cysA *genes do not form an operon in plastids, so we assume the CysT protein's double abundance over the CysA protein. It conforms to our hypothesis.

In *E. coli,* CysT and CysW are transmembrane domains with similar structure. Their genes belong to the same operon with the sulfate transport subunit *cysPTWAM*; however, *cysW* is absent in plastids. We hypothesize that in plastids the CysW subunit is functionally replaced by another copy of CysT, and the *cysT* mRNA concentration is twice as high compared to *cysA* mRNA. This hypothesis is indirectly supported by the fact that *cysT* and* cysA *are not included in one operon in plastids, and thus their mRNA expression levels may differ considerably.

## Figures and Tables

**Table 1:** CysA and CysT proteins encoded in plastids of the Viridiplantae and in the cyanobacterium *Synechocystis* sp. PCC 6803.

Species	DNA locus	GC, %	CysA	CysT
*Synechocystis* sp. PCC 6803	NC_017277.1	49	YP_005652904.1	YP_005652902.1
*Nephroselmis olivacea *	NC_000927.1	42	NP_050872.1	NP_050928.1
*Pycnococcus provasolii *	NC_012097.1	40	None	YP_002600832.1
*Bryopsis hypnoides *	NC_013359.1	33	YP_003227066.1	YP_003227041.1
*Chlorella variabilis *	NC_015359.1	34	YP_004347745.1	YP_004347762.1
*Chlorella vulgaris *	NC_001865.1	32	NP_045832.1	NP_045890.1
*Coccomyxa subellipsoidea *	NC_015084.1	51	YP_004222028.1	YP_004221988.1
*Helicosporidium *sp.	NC_008100.1	27	None	YP_635918.1
*Leptosira terrestris *	NC_009681.1	27	YP_001382174.1	YP_001382135.1
*Parachlorella kessleri *	NC_012978.1	30	YP_003058285.1	YP_003058340.1
*Mesostigma viride *	NC_002186.1	30	NP_038429.1	NP_038441.1
*Chlorokybus atmophyticus *	NC_008822.1	36	YP_001019096.1	YP_001019170.1
*Zygnema circumcarinatum *	NC_008117.1	31	YP_636486.1	YP_636569.1
*Anthoceros formosae *	NC_004543.1	33	NP_777407.1	NP_777462.1
*Aneura mirabilis *	NC_010359.1	41	Pseudogene	Pseudogene
*Marchantia polymorpha *	NC_001319.1	29	NP_039293.1	NP_039346.1
*Ptilidium pulcherrimum *	NC_015402.1	33	Pseudogene	Pseudogene

**Table 2:** The genomic context of the *cysA* and *cysT* genes in plastids of the Viridiplantae. The symbol “&” designated the lack of a bacterial-type promoter, “!” means that the intergenic region is very short, “*P”* designates the presence of a bacterial type promoter, “∗” designates a pseudogene, “()” marks an opposite direction, and “#” designates prediction of a conserved site.

Species	*cysA *	*cysT *
*Nephroselmis olivacea *	(*trnE*)*-*&*-cysA-*(*psbZ*)	*rpl32-*&*-cysT-ycf1 *
*Pycnococcus provasolii *	None	*trnP-*&*-cysT-ycf1 *
*Bryopsis hypnoides *	*ccsA-*&!*-cysA-psbB *	*rpl12-*&*-cysT-*(*rpoA*)
*Chlorella variabilis *	*accD-#P-cysA-*(*trnT1*)	*rpl32-#P-cysT-ycf1 *
*Chlorella vulgaris *	*accD-*&*-cysA-*(*trnT*)	*rpl32-##P-cysT-orf819 *
*Coccomyxa subellipsoidea *	*accD-###P-cysA-*(*trnN*)	*rpl32-##P-cysT-ycf1 *
*Helicosporidium *sp.	None	*ftsH-##P-cysT-ycf1 *
*Leptosira terrestris *	*orf96-*&*-cysA-rbcL *	*orf67-*&*-cysT-*(*trnC*)
*Parachlorella kessleri *	*accD-#P-#P-cysA-*(*trnT*)	*rpl32-#P-#P-cysT-ycf1 *
*Mesostigma viride *	(*trnE*)*-#P-cysA-*(*trnT*)	*rpl32-#P-#P-cysT-ycf1 *
*Chlorokybus atmophyticus *	(*trnR*)*-*&*-cysA-*(*trnT*)	(*rpl32*)*-P-cysT-ycf1 *
*Zygnema circumcarinatum *	(*trnE*)*-#P-cysA-trnT *	*trnV-*&*-cysT-*(*rpl21*)
*Anthoceros formosae *	*trnE-#P-#P-#P-cysA-trnT *	*rpl32-#P-cysT-*(*trnP*)
*Aneura mirabilis *	(*trnE*)*-cysA∗-trnT *	*rpl32-*&*-cysT∗-*(*trnP*)
*Marchantia polymorpha *	(*trnE*)*-P-mbpX-trnT *	*rpl32-P-cysT-trnP∗ *
*Ptilidium pulcherrimum *	(*trnE*)*-cysA∗-trnT *	*rpl32-cysT∗-trnL *

## References

[1] Tartar A, Boucias DG, Adams BJ, Becnel JJ (2002). Phylogenetic analysis identifies the invertebrate pathogen *Helicosporidium* sp. as a green alga (Chlorophyta). *International Journal of Systematic and Evolutionary Microbiology*.

[2] de Koning AP, Keeling PJ (2006). The complete plastid genome sequence of the parasitic green alga *Helicosporidium* sp. is highly reduced and structured. *BMC Biology*.

[3] Pombert J-F, Keeling PJ (2010). The mitochondrial genome of the entomoparasitic green alga *Helicosporidium*. *PloS ONE*.

[4] Sadovskaya TA, Seliverstov AV (2009). Analysis of the 5°-Leader regions of several plastid genes in protozoa of the phylum apicomplexa and red algae. *Molecular Biology*.

[5] Tajima N, Sato S, Maruyama F (2011). Genomic structure of the cyanobacterium *synechocystis* sp. PCC 6803 strain GT-S. *DNA Research*.

[6] Thelwell C, Robinson NJ, Turner-Cavet JS (1998). An SmtB-like repressor from *synechocystis* PCC 6803 regulates a zinc exporter. *Proceedings of the National Academy of Sciences of the United States of America*.

[7] Takahashi H, Saito K (1996). Subcellular localization of spinach cysteine synthase isoforms and regulation of their gene expression by nitrogen and sulfur. *Plant Physiology*.

[8] Thomsen-Zieger N, Schachtner J, Seeber F (2003). Apicomplexan parasites contain a single lipoic acid synthase located in the plastid. *FEBS Letters*.

[9] Kredich NM (1992). The molecular basis for positive regulation of cys promoters in *Salmonella typhimurium* and *Escherichia coli*. *Molecular Microbiology*.

[10] Lynch AS, Tyrrell R, Smerdon SJ, Briggs GS, Wilkinson AJ (1994). Characterization of the CysB protein of *Klebsiella aerogenes*: direct evidence that N-acetylserine rather than O-acetylserine serves as the inducer of the cysteine regulon. *Biochemical Journal*.

[11] Zverkov OA, Rusin Yu L, Seliverstov AV, Lyubetsky VA (2013). Study of direct repeats in micro evolution of plant mitochondria and plastids based on protein clustering. *Moscow University Biological Sciences Bulletin*.

[12] Zverkov OA, Seliverstov AV, Lyubetsky VA (2012). Plastid-encoded protein families specific for narrow taxonomic groups of algae and protozoa. *Molecular Biology*.

[13] Seliverstov AV, Lysenko EA, Lyubetsky VA (2009). Rapid evolution of promoters for the plastome gene *ndhF* in flowering plants. *Russian Journal of Plant Physiology*.

[14] Lyubetsky VA, Rubanov LI, Seliverstov AV (2010). Lack of conservation of bacterial type promoters in plastids of Streptophyta. *Biology Direct*.

[15] Lyubetsky VA, Zverkov OA, Rubanov LI, Seliverstov AV (2011). Modeling RNA polymerase competition: the effect of *σ*-subunit knockout and heat shock on gene transcription level. *Biology Direct*.

[16] Lyubetsky VA, Zverkov OA, Pirogov SA, Rubanov LI, Seliverstov AV (2012). Modeling RNA polymerase interaction in mitochondria of chordates. *Biology Direct*.

[17] Homann A, Link G (2003). DNA-binding and transcription characteristics of three cloned sigma factors from mustard (*Sinapis alba* L.) suggest overlapping and distinct roles in plastid gene expression. *European Journal of Biochemistry*.

[18] Lyubetsky VA, Seliverstov AV (2003). Some algorithms related to finite groups. *Information Processes*.

[19] Su Z, Olman V, Mao F, Xu Y (2005). Comparative genomics analysis of NtcA regulons in cyanobacteria: regulation of nitrogen assimilation and its coupling to photosynthesis. *Nucleic Acids Research*.

[20] Thompson JD, Gibson TJ, Plewniak F, Jeanmougin F, Higgins DG (1997). The CLUSTAL X windows interface: flexible strategies for multiple sequence alignment aided by quality analysis tools. *Nucleic Acids Research*.

[21] Wickett NJ, Forrest LL, Budke JM, Shaw B, Goffinet B (2011). Frequent pseudogenization and loss of the plastid-encoded sulfate-transport gene cysA throughout the evolution of liverworts. *American Journal of Botany*.

[22] Lyubetsky VA, Seliverstov AV, Zverkov OA (2013). Elaboration of the homologous plastid-encoded protein families that separate paralogs in magnoliophytes. *Mathematical Biology and Bioinformatics*.

[23] Koonin EV, Wolf YI, Aravind L (2000). Protein fold recognition using sequence profiles and its application in structural genomics. *Advances in Protein Chemistry*.

[24] Punta M, Coggill PC, Eberhardt RY (2012). The Pfam protein families database. *Nucleic Acids Research*.

[25] Wilson RJM, Rangachari K, Saldanha JW (2003). Parasite plastids: maintenance and functions. *Philosophical Transactions of the Royal Society B*.

